# Supplementary material for: Innovative photodynamic therapy using rose bengal for the treatment of human melanoma
Source: Mol Ther Oncol. 2026 May 21;34(2):201241. doi: 10.1016/j.omton.2026.201241 (PMC13254722; doi:10.1016/j.omton.2026.201241)
Supplement: Document S1. Figures S1 and S2 [file mmc1.pdf]

OMTON, Volume *34*

Supplemental information

Innovative photodynamic therapy using rose bengal for the treatment of human melanoma

Marie Boileau, Anthony Lefebvre, Smail Marhfor, Meriem Kali Mansouri, Pascal Deleporte, Guillaume Paul Grolez, Anne-Sophie Dewalle, Olivier Morales, Nadira Delhem, and Laurent Mortier

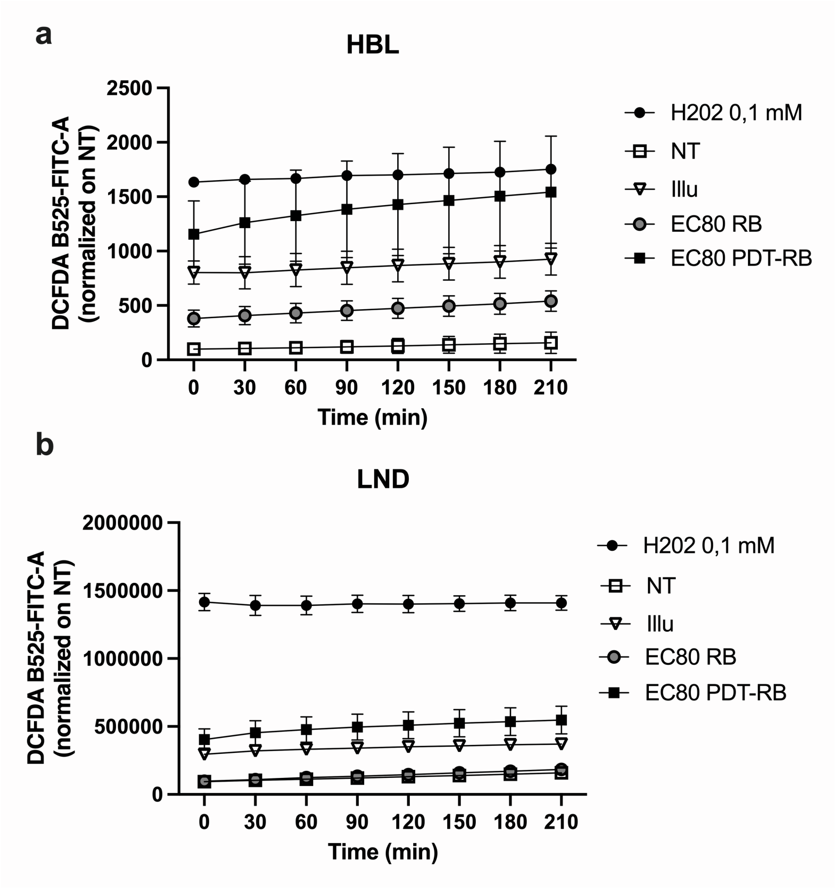

Figure S1

ROS production kinetics were assessed every 30 minutes over a period of 3.5 hours **(a for HBL and b for LND).** ROS production was constant over time. NT: Non treated cells, Illu: illuminated alone without Rose Bengal, RB: Rose Bengal, PDT-RB: cells incubated with RB and illuminated, H2O2 : positive control

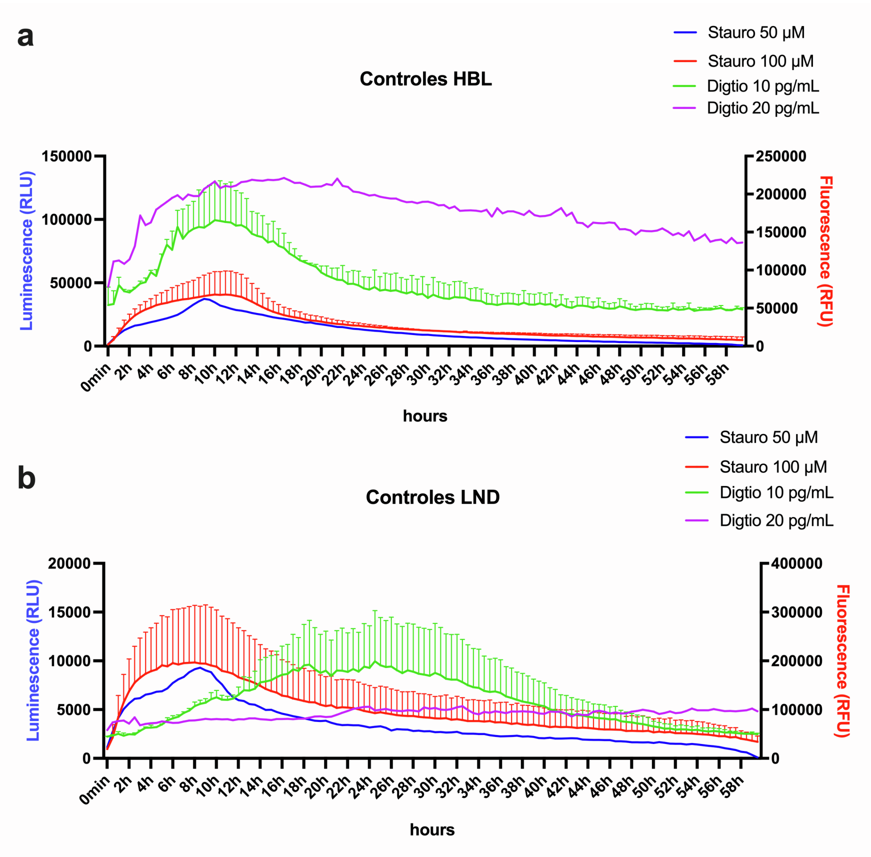

Figure S2

Kinetic profiles of Phosphatidyl Serine exposure reflecting apoptosis (luminescence) and loss of membrane integrity reflecting necrosis (fluorescence) over time according to the concentration of RB. Luminescence and Fluorescence signal were collected by sequential measurements of the same plate over the 60h time course. Staurosporine was used as a positive control of apoptosis and Digitonin as a positive control of necrosis **(a for HBL and b for LND)**
