# Supplementary material for: Innovative photodynamic therapy using rose bengal for the treatment of human melanoma
Source: Mol Ther Oncol. 2026 May 21;34(2):201241. doi: 10.1016/j.omton.2026.201241 (PMC13254722; doi:10.1016/j.omton.2026.201241)
Supplement: Document S2. Article plus supplemental information [file mmc2.pdf]

Molecular Therapy
Oncology
Original Article

Innovative photodynamic therapy using rose bengal for the treatment of human melanoma

Marie Boileau,[1,2,4] Anthony Lefebvre,[1,4] Smail Marhfor,[1,3] Meriem Kali Mansouri,[1] Pascal Deleporte,[1] Guillaume Paul Grolez,[1] Anne-Sophie Dewalle,[1] Olivier Morales,[1] Nadira Delhem,[1,5] and Laurent Mortier[1,2,5]

[1]University Lille, CHU Lille Inserm, U1366 – CRC Lille CNRS UMR 9020, ONCOTHAI - Assisted Laser Therapy and Immunotherapy for Oncology, 59000 Lille, France; [2]Service de Dermatologie, Hôpital Claude Huriez CHU de Lille, 59000 Lille, France; [3]University Grenoble Alpes, CEA, LETI, 38000 Grenoble, France

Rose Bengal (RB), a xanthene dye with established antitumor and immunomodulatory properties, is a cytotoxic molecule evaluated in human melanoma through phase 2 clinical trials. RB demonstrates intrinsic therapeutic activity; however, its clinical application may be limited at high doses because of potential side effects, including photosensitivity. In this context, and given that RB also functions as a photosensitizer (PS), our objective was to enhance its efficacy by introducing a photodynamic therapy (PDT) approach using green light (550 nm) excitation. PDT relies on activation of a PS in the presence of oxygen, generating reactive oxygen species (ROS) that induce tumor cell death and stimulate immune responses. In terms of relative efficacy, PDT-RB was 121 and 40 times more effective than RB alone in HBL and LND melanoma cell lines, respectively. This reduced the RB concentration from 492 μM to 32 μM for HBL and from 1,043 μM to 74 μM for LND, achieving 80% cell death. This combination of light and low RB concentrations enhances ROS production and induces necrosis in metastatic melanoma cells. Moreover, PDT-RB preserves PBMC proliferation compared with high-dose RB, suggesting reduced immunotoxicity and potential immunogenic properties, supporting further investigation in more advanced models for future validation.

INTRODUCTION

Melanoma is an aggressive skin cancer with metastatic potential, particularly in the cutaneous region. This cancer is highly immunogenic, partly due to a high mutational load linked to its UV-induced nature.[1]

In the case of metastatic disease, first-line treatment is based on immunotherapies and targeted therapies. The choice of treatment depends on the *BRAF* mutation status (present in 40%–50% of patients), the metastatic load and the patient's general condition.[2]

The therapeutic tools available are immunotherapies with immune checkpoint inhibitors and targeted therapies with MPAKinase inhibitors.[2] Approximately 35%–60% of patients show a RECIST response to anti-PD-1 immunotherapy; however, 40%–65% showed minimal or no RECIST response at baseline, and nearly one-third of responders develop acquired resistance.[2] Some patients do not respond to treatment and can present a disabling loco-regional evolution. It is therefore necessary to continue research into new therapeutic strategies or to find potentiating treatments that will overcome these therapeutic limitations. Strategies aimed at improving the response to conventional treatments are currently in the pipeline, including strategies involving intratumoral injection and the induction of an immune response following injection. For example, trials combining immunotherapies with mRNA vaccination[3] or intralesional injection of cytotoxic agent such as Rose Bengal (RB) in its clinical form, PV-10.[4]

Indeed, RB is a xanthene dye, is a hydrophilic anionic sensitizer with better solubility in aqueous media, potentially accumulating in melanoma cells. Initially used as an ophthalmic diagnostic agent, it has recently attracted growing interest in oncology, particularly for its ability to induce apoptosis and tumor necrosis.[4] The injected derivative of RB diluted at 10% in NaCl, PV-10, has shown objective responses ranging from 51% to 69% in clinical trials on refractory metastatic melanoma without light.[5–8] However, photosensitivity to daylight was noted with the appearance of erythroderma or perilesional blisters.[6,9] These significantly associated with a tumor response in both injected and non-injected lesions. This suggests its immunostimulatory properties[6] but also its photosensitizing properties. RB could therefore be a photosensitizing agent that can be used in photodynamic therapy (PDT) for melanoma.

PDT is a common non-invasive treatment based on the association of 3 elements, a photosensitive molecule named photosensitizer (PS), a light with PS specific wavelength and intracellular oxygen

Received 16 December 2025; accepted 19 May 2026;
https://doi.org/10.1016/j.omton.2026.201241.

[4]These authors contributed equally

[5]These authors contributed equally

Correspondence: Marie Boileau, Department of Dermatology, CHU of Lille, Lille, France.
E-mail: marie.boileau@chu-lille.fr
Correspondence: Guillaume Grolez, Cancer Research Center of Lille U1366 OncoLille Institute, ONCOTHAI - Assisted Laser Therapy and Immunotherapy for Oncology, 59000 Lille, France.
E-mail: guillaume.grolez@inserm.fr

© 2026 The Authors.
Published by Elsevier Inc. on behalf of The American Society of Gene and Cell Therapy.
This is an open access article under the CC BY license (http://creativecommons.org/licenses/by/4.0/).

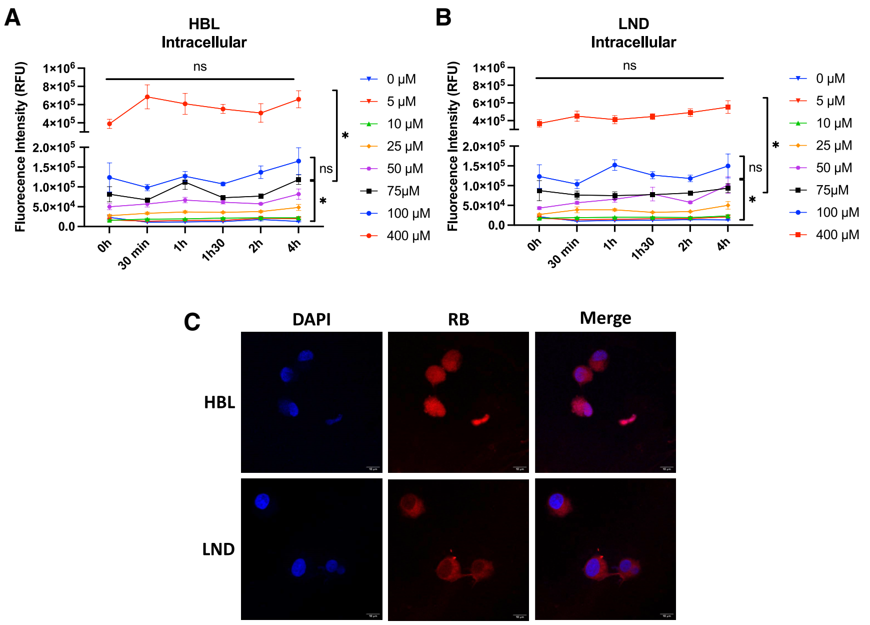

Figure 1. Rose Bengal incorporation in human melanoma cell lines HBL and LND

Incorporation study of RB in HBL and LND cell lines. (A and B) Intracellular fluorescence of RB according to RB concentration and incubation time respectively in HBL and LND melanoma cell lines. (C) Confocal microscopy images showing RB intracellular incorporation with nuclei stained with DAPI (blue) after 2 h of treatment in HBL and LND cells. Scale bars are 10 µm.

present in the targeted cells. Once the PS is administrated, it will incorporate inside the pathological tissues. After a light stimulation, the PS switch from a basic singlet energy state to an excited state. A part of the energy is converted into fluorescence and the remaining energy will make the PS enter into an unstable triplet excited state. Return to baseline lead to oxidative stress and reactive oxygen species (ROS) production inducing tumor cells death. This PDT tumor cell death conduct to the liberation of damage-associated molecular patterns (DAMPs) recognized by the immune system, which in turn induces the activation of both innate and adaptive immune responses against tumor cells.[10–12] This selective approach has the advantage of limiting adverse effects on healthy tissue. PDT is commonly used in dermatology to treat squamous cell carcinoma *in situ*, superficial basal cell carcinoma and actinic keratosis.[13] Nowadays, PDT has also demonstrated really good results in the fields of oncology.[14–16] Although PDT works well in other pathologies, its application in melanoma has sometimes met with lack of efficacy. The presence of melanin and organelles such as melanosomes has been reported to reduce PDT efficacy.[17] The most commonly used PSs, such as 5-ALA, have shown limited efficacy in melanoma.[17] Development of new PSs and strategies is thus needed.

Although several studies have demonstrated the efficacy of light-activated RB against various tumor types, its potential in the treatment of human melanoma remains largely unexplored. As the molecule is already effective at high doses in melanoma, it could be optimized by adding light to achieve a PDT effect. Forms coupled with nanoparticles or amphiphilic peptides have shown signs of efficacy with a reduction in melanoma proliferation *in vitro* or *in vivo*.[4,18,19] It is therefore crucial to evaluate its phototoxic effects and mechanisms of action in this context. Our main hypothesis was that adding light to RB would improve its efficacy through a PDT effect, reducing the doses of RB required to achieve an equivalent anti-tumor effect.

In addition, we explored whether PDT-RB could exhibit immunologically relevant effects. The aim of this study was to evaluate the efficacy and underlying mechanisms of PDT-RB in a human melanoma *in vitro* model, as well as its impact on PBMC proliferation, to assess its potential as a complementary therapeutic strategy.

RESULTS

RB incorporates into melanoma cells

We first verified the ability of RB to incorporate into two human metastatic melanoma cell lines HBL and LND. To do this, we performed fluorometric measurements at increasing concentrations over time. We showed that intracellular fluorescence, and therefore incorporation, was dose and time dependent. This incorporation was immediate, constant, and stable over time (Figures 1A and 1B). We confirmed intracellular incorporation by confocal microscopy, showing essentially cytoplasmic incorporation, as expected (Figure 1C).

RB at high dose is effective against metastatic melanoma cells

We then evaluated the impact RB on the viability of metastatic melanoma cell lines. As expected, we showed that high doses of RB induce a decrease in the viability of melanoma cell lines, in this case starting at 100 µM for HBL and 400 µM for LND (Figure 2, a for HBL and b for LND). We confirmed, through a test measuring LDH release levels after RB treatment, that the low doses of RB were not toxic in the absence of light, unlike high concentrations (Figures 2C and 2D).

High doses of RB are detrimental to immune cells

Since we observed a toxic effect of high RB concentration on cancer cell, we sought to analyze this effect on immune cells; and hope to confirm a benefit of reducing RB doses to preserve the immunological response. To do this, we first performed peripheral blood mononuclear cells (PBMCs) proliferation kinetics at 24, 96, and 120 h after treatment with increasing concentrations of RB in non-activated or

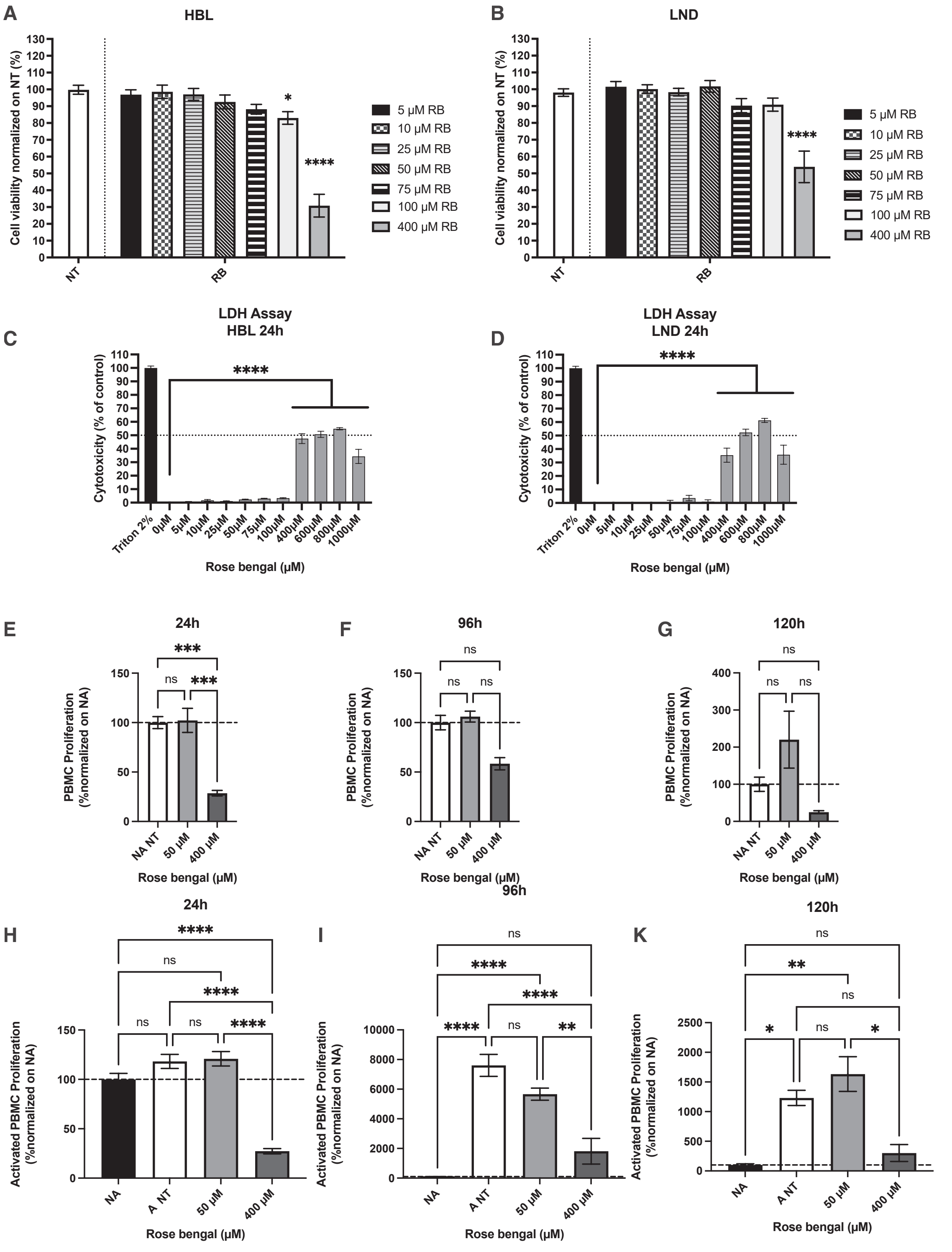

(legend on next page)

PHA-activated conditions (Figure 2). On non-activated PBMCs, we showed that RB alone at high concentrations (here 400 μM) induced a significant decrease in proliferation at 24 h, interestingly it was not the case for low concentrations (50 μM here) (Figures 2E, 2F, and 2G). After PHA activation, we observed a significant decrease in PBMCs proliferation, which was maintained over time with these high concentrations (Figures 2H, 2I, and 2J). These results indicate that it would also be beneficial to reduce RB concentrations, in order to avoid a decline in PBMCs proliferation, as this would be detrimental to the induction of an immune response.

RB-PDT increases the efficacy of RB on metastatic melanoma cells

When green light at 550 nm at a dose of 0.3 J/cm^2 was added to RB (PDT-RB), a significant decrease in viability was observed at lower concentrations of RB, starting at 25 μM for HBL and 75 μM for LND. This decrease in viability under PDT-RB conditions was significant and dose dependent in both cell lines (Figures 3A and 3B). An observation was made of a discrepancy in the efficacy of the two human melanoma cell lines. We then wanted to evaluate the effect of escalating dose of green light on human melanoma cell lines. Regardless of the light dose ranging from 0.3 J/cm^2 to 1.22 J/cm^2, there was no impact of green light alone at 550 nm on the viability of HBL and LND (Figures 3C and 3D). However, at low RB concentrations (here 25 μM), a dose-dependent decrease in viability was observed with increasing irradiation (Figures 3B and 3D). All of these results allowed us to establish an isobologram for the two cell lines illustrating the synergy between green light and RB (Figures 3E and 3F). This allowed us to establish the RB concentrations for further experiments to achieve viability reductions of 20%, 50%, and 80% for the two human melanoma cell lines with green light at 0.3 J/cm^2 (table in Figure 2G). In terms of relative efficacy (RB viability/PDT-RB viability) for each line, PDT-RB is approximately 121 times and 40 times more effective than RB alone for HBL and LND, respectively, with lower viability indicating greater efficacy. This means that PDT-RB is more effective than RB alone in both lines, with an effect more pronounced in the HBL line than in the LND line.

PDT-RB induces oxidative stress in metastatic melanoma cells

PDT induces cell death by inducing ROS. To this end, we first wanted to confirm by flow cytometry, measuring the fluorescence emitted by 2′,7′-dichlorofluorescein diacetate (DCFDA), that low concentrations of RB combined with green light under PDT-RB conditions, resulting in an 80% decrease in viability (EC80[PDT-RB]), were capable of inducing ROS. The results show that PDT-RB at EC80 induces ROS production, whereas RB alone at EC80 does not (Figures 4A–4D). In order to test a wider range of concentrations and explore ROS kinetic, we observed that the mean fluorescence increased with concentration under PDT-RB conditions. This finding indicates a dose-dependent enhancement in ROS production under PDT-RB conditions. However, this was not the case for increasing concentration of RB out of light activation (Figures 4E and 4F). We then used the same method to explore the kinetics of ROS production over 210 min. ROS production was immediate and constant over time (Figures S1A and S1B).

Necrosis is the principal mechanism of cell death during PDT-RB

To detect dose- and time-dependent apoptosis and necrosis over 60 h post-treatment, the cell death mechanism induced by PDT was studied. Green light alone at 0.3 J/cm^2 did not induce loss of membrane integrity (Figures 5C and 5D), the luminescence (apoptosis) and fluorescence (necrosis) profiles were comparable to those of the untreated conditions over time (Figures 5A and 5B). We found expected results of cell death at EC80[RB] mainly by necrosis with a fluorescence peak around 20 h for the two melanoma lines. Low doses of RB optimized using green light, resulting in an 80% decrease in viability EC80[PDT-RB], also induced a fluorescence peak around 20 h in both cell lines (Figures 5E and 5F). This necrosis was dose-dependent for both cell lines (Figures 5J and 5I). Apoptosis was not detected at any concentration for the two lines, either with RB alone or under PDT-RB conditions at increasing concentrations. (Figures 5E, 5F, 5K, 5G, 5H, and 5L). Moreover, necrosis is well known to have immunostimulatory properties.

PDT-RB induces an indirect immune response

To investigate potential immune-related effects of PDT-RB, we treated PBMCs activated with anti-CD3 and anti-CD28 antibodies, mainly used to stimulate T lymphocytes, with HBL and LND melanoma cell line supernatants harvested 24 h after PDT-RB treatment. We then evaluated PBMCs proliferation over time using the same assay. We observed a significant increase in the proliferation of activated PBMCs after treatment with the supernatant of metastatic melanoma cells treated with PDT-RB at 72 h (Figure 6A with HBL supernatant and 6B with LND supernatant) and 120 h (Figure 6C with HBL supernatant and 6D with LND supernatant).

These findings suggest that PDT-RB induces the release of soluble factors capable of enhancing immune cell proliferation. Overall, photodynamic activation of RB may promote immune-compatible effects while enabling dose reduction.

DISCUSSION

The findings of this study indicate that PDT-RB, involving the application of green light at a wavelength of 550 nm with an intensity of

Figure 2. Rose Bengal effect on human melanoma cell lines and PBMC

(A and B) Respective cell viability of HBL and LND cells 24 h after RB treatment at different concentration. Two-way ANOVA statistical test was performed; all quoted p values are two-sided with ****$p < 0.0001$. (C and D) Rose bengal cytotoxicity on HBL and LND cells 24 h after treatment. Results are presented as mean ± SEM of 3 independent experiments, expressed in percentage (%) of cytotoxicity and compared to positive control (Triton). One-way ANOVA statistical test was performed; all quoted p values are two-sided with ****$p < 0.0001$. PBMCs proliferation after 24, 96 and 120 h post RB incubation during 2 h in non-activated condition (E–G) and activated with 2 μM of PHA condition (H–J). Result of 3 independent experiments. One-way ANOVA statistical test was performed, with *$p < 0.05$, **$p < 0.01$ and ***$p < 0.001$.

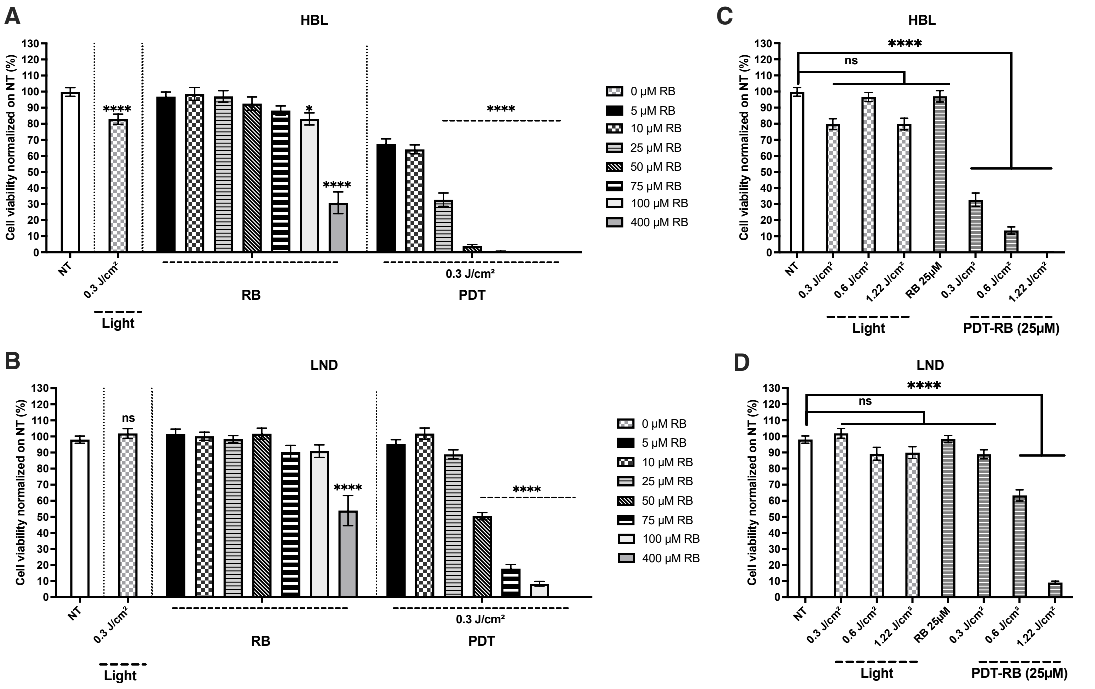

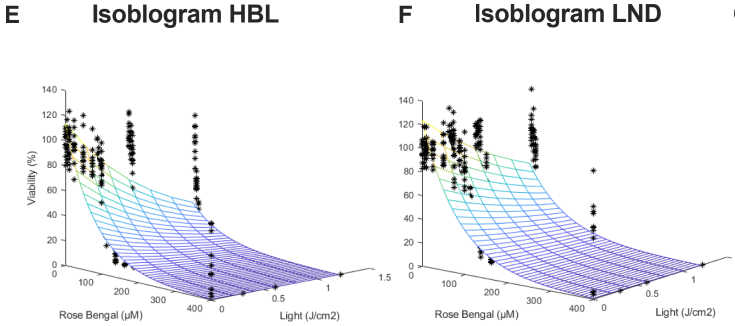

G

	HBL		LND	
	RB	PDT-RB	RB	PDT-RB
EC20	134 µM	13 µM	363 µM	33 µM
EC50	309 µM	22 µM	708 µM	50 µM
EC80	492 µM	32 µM	1043 µM	74 µM
EC100	916 µM	96 µM	1237 µM	278 µM

Figure 3. Synergy effect of Rose Bengal associated to green light against human metastatic melanoma cell lines

(A and B) Respectively represent the cell viability of HBL and LND cells 24 h after RB-PDT at 0.3J/cm^2. Results are presented as mean ± SEM of 3 independent experiments. Two-way ANOVA statistical test was performed, all quoted p values are two-sided with ****$p < 0.0001$. (C and D) HBL and LND cell viability 24 h after PDT treatment at several light doses. Results are represented as mean ± SEM of 3 independent experiments, expressed in percentage (%) compared to None Treated (NT) cells condition ($n = 3$). Kruskal-Wills statistical test was performed, all quoted ****$p < 0.0001$. (E and F) Isobologram representing interaction between Rose Bengal (µM) and green light (J/cm^2) respectively for HBL and LND cell lines. The axes indicate the concentrations of Rose Bengal and doses of green light required to achieve viability decrease when administered alone or in combination. (G) Effective concentration (EC20, EC50, EC80, and EC100) values were calculated from the viability curves for both cell lines.

0.3 J/cm^2, can enhance the efficacy of RB in human melanoma treatment. Indeed, RB has been found to incorporate into human melanoma cell lines. The substance has been demonstrated to induce a decrease in the viability of human melanoma cell lines at high doses in the absence of other factors. However, these elevated concentrations do not enhance immunogenicity, as evidenced by the observed decline in PBMCs proliferation. This phenomenon is not observed in the context of low concentrations of RB. We demonstrated that the addition of light to low concentrations of RB (PDT-RB) reduces the viability of human metastatic melanoma cell lines while optimizing the immunological response by inducing PBMCs proliferation.

RB indirect immunostimulatory properties are known in mouse models of melanoma and other types of cancer. This anti-tumor effect in melanoma was confirmed in a murine melanoma model in mice treated with intra-lesional injection of PV-10, the clinical form of RB in NaCl 0, 9% at 10% used in trial.[20]

It was shown in a mouse model of B16 melanoma injected into C57BL/6 mice that treatment of the subcutaneous lesion with an intralesional injection of PV-10 (IL PV-10) resulted in regression of the injected lesion as well as distant lung metastases. IL PV-10 induced increased production of tumor-specific IFN-gamma. In addition,

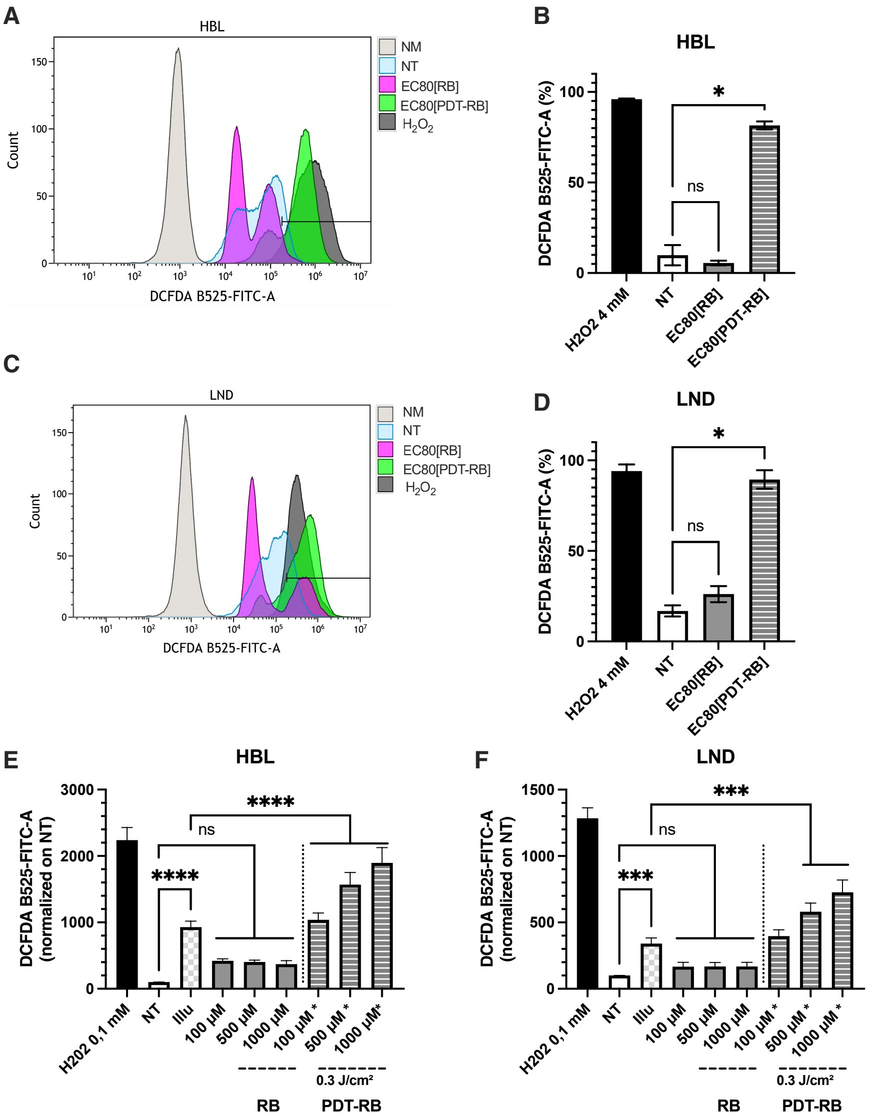

Figure 4. ROS generation in human metastatic melanoma cell lines treated with PDT-RB

(A and C) Representative overlay of ROS production analyzed by flow cytometry in HBL and LND cells. (B and D) respectively represent DCFDA MFI histograms profiles from 3 independent experiments for HBL and LND cells. One-way ANOVA statistical test was performed, with *p < 0.005 (n = 3). (E and F) Ros production in both HBL and LND after PDT-RB assessed by fluorescence spectroscopy. Results are presented as mean ± SEM of 3 independent experiments. Two-way ANOVA statistical test was performed, all quoted p values are two-sided with ***p < 0.001 ****p < 0.0001.

they observed a significant increase in the lysis of B16 cells by isolated T cells after treatment with IL PV-10, indicating a tumor specific immune response developed through PDT.[20]

In an immunocompetent mouse model of colon cancer, tumors were shown to regress after treatment with RB, and RB was shown to induce cell death through G2/M arrest and predominant necrosis.

Colon cancer cells treated with RB exhibit distinct features of immunogenic cell death, including increased expression of calreticulin and heat shock protein 90 on the cell surface, decreased intracellular ATP, and release of HMGB1.[21] Liu et al. showed that RB injections can lead to tumor cell necrosis and HMGB1 release, with increased dendritic cell (DC) infiltration into draining lymph nodes and activation of tumor-specific T cells. Treatment of DCs with tumor supernatants increased the ability of DCs to stimulate T cell proliferation, and blocking HMGB1 in supernatants suppressed DC activity. In addition, increased levels of HMGB1 were measured in the serum of melanoma patients treated with intralesional RB injection. These results confirm the role of RB in activating DCs at the site of tumor necrosis to induce a systemic antitumor immune response.[22] Our *in vitro* results suggest that PDT-RB not only enhances direct tumor cell killing but may also modulate immune-related responses through the release of soluble factors. Indeed, supernatants from PDT-RB-treated melanoma cells significantly increased the proliferation of activated PBMCs compared to untreated controls. This observation is consistent with the notion that PDT can induce the release of danger-associated molecular patterns and other immunologically active mediators. Importantly, in contrast to high-dose RB alone, PDT-RB appears to preserve or enhance immune cell proliferation, supporting a reduced immunotoxic profile. While these findings do not demonstrate a direct antitumor immune response, they highlight the potential of PDT-RB to create an immune-compatible environment. Further studies will be required to characterize the nature of the released factors and to evaluate their relevance *in vitro* and *in vivo*.[11]

In a study of the antitumor response of PV-10 (RB) associated with PD-1/PD-L1 pathway blockade, it was shown that mice bearing B16 or M05 melanoma tumors treated with PV-10 had decreased tumor growth and increased tumor antigen-specific CD8+ T cells and interferon gamma. Depletion of regulatory T cells (Tregs) in combination with ILPV-10 and anti-PD-1 resulted in an increase in the antitumor effect.[22] Injections of DCs coupled with RB in a mouse model of lung cancer showed, in addition to a reduction in subcutaneous tumor growth and lung metastases, a significant increase in TNF-α production by $CD8^+$ T cells. These effects were closely linked to the induction by RB of calreticulin expression, increased infiltration of effector immune cells (i.e., $CD4^+$ T cells, $CD8^+$, and memory cells) and a

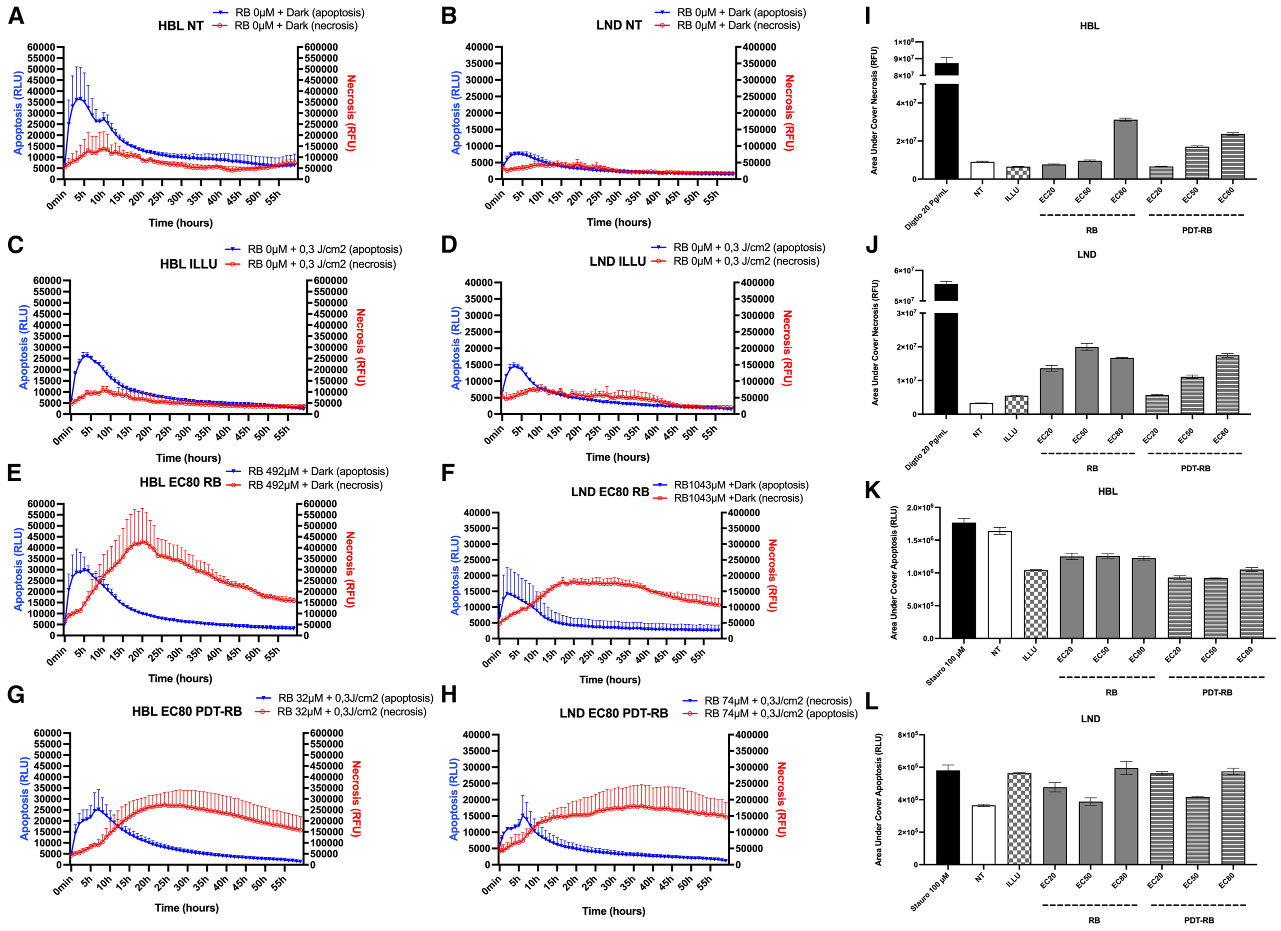

Figure 5. Types of cell death induced by RB and PDT-RB on human metastatic melanoma cell lines

(A–H) Kinetic profiles of phosphatidyl serine exposure reflecting apoptosis (luminescence) and loss of membrane integrity reflecting necrosis (fluorescence) over time according to the concentration of RB and association with light. Luminescence and fluorescence signal were collected by sequential measurements of the same plate over the 60 h time course post-PDT. (I–L) Area under curves (AUC) were calculated from each curve. Results are presented as mean ± SEM of 2 independent experiments ($n = 2$). NT, non-treated cells; Illu, illuminated alone without Rose Bengal; RB, Rose Bengal; PDT-RB, cells incubated with RB and illuminated.

decrease in the accumulation of immunosuppressive cells (i.e., Tregs, macrophages, and myeloid-derived suppressor cells [MDSCs]) in the tissue. This study suggested the immunostimulatory potential of RB in combination with immature DC vaccinations.[23]

As we saw in the introduction, RB used in the form of PV-10 in intralesional injections for the treatment of metastatic melanoma, shows promising results, inducing a local and distant tumor response that is likely immune-mediated. However, its use has several limitations. First, phototoxicity is a major problem.[6,24] Bullous lesions have been observed near the injection sites. In addition, cases of grade III-IV phototoxicity have been described in connection with probable systemic passage of the drug, inducing photosensitivity to daylight. Furthermore, intralesional injection is often painful, particularly for patients with multiple lesions.[9] Systemic passage of RB after intralesional injection is certain (documented exertion) with organic toxicity still poorly assessed at this stage.[25]

To overcome these limitations, RB could be optimized. Several approaches could be explored, such as developing topical formulations and/or reducing doses, while maintaining therapeutic efficacy by optimizing its photosensitizing properties.

In this regard, we wanted to confirm the role of RB in PDT as a PS. The photochemical properties of RB are mainly due to its halogenated xanthene core, which enables the absorption of photons and the generation of ROS.

The efficacy of PDT-RB has been explored in other *in vitro* models of breast cancer MCF7 and the HeLa line.[26,27] Preliminary studies aimed at explaining the phototoxicity of RB induced by UV and visible light conducted by Srivastav et al. suggest efficacy in A375 melanoma. However, the wavelength used here was not optimized for the RB spectrum, and this study aimed to explain the phototoxicity of the molecule not its potential use as an anti-cancer drug in PDT.[28]

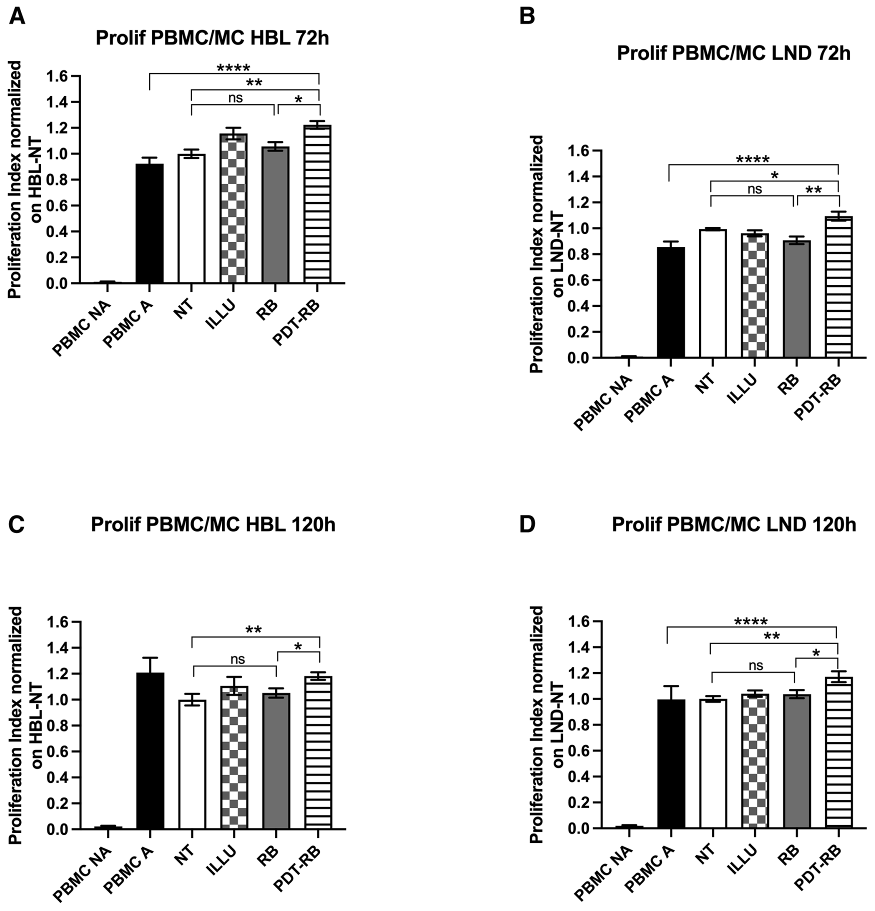

Figure 6. Evaluation of immunologic response with PDT-RB

(A–D) Proliferation of activated PBMCs (anti-CD3 – anti-CD28 cocktail) in co-culture with conditioned media derived from HBL (A and C) and LND (B and D) cell lines, respectively, after 72 h and 120 h of culture. Results are presented as mean ± SEM of 4 independent experiments, one-way ANOVA statistical test was performed with $*p < 0.05$, $**p < 0.001$ being considered statistically significant. NT, non-treated cells; Illu, illuminated alone without Rose Bengal; RB, Rose Bengal; PDT-RB, cells incubated with RB and illuminated.

PDT is already commonly used in dermatology for the treatment of skin cancers such as superficial basal cell carcinoma and squamous cell carcinoma *in situ*.[13] This treatment is interesting for its immunostimulatory anti-cancer properties. Melanoma is a highly immunogenic cancer. However, few studies have looked at PDT in melanoma. We therefore chose to use a molecule that is known to be effective in melanoma but has several limitations, and to optimize it using PDT.

We confirmed the results of intrinsic cytotoxic properties known in the literature for melanoma. Indeed, Mousavi et al. showed that RB could induce cell death in different melanoma cell lines (Me4405, SKMel 28) but not in fibroblast cells. This toxicity was mainly induced by non-apoptotic cell death.[29] We add to this knowledge the notion of direct immunolo-toxicity of RB through a decrease in PBMC proliferation. This finding serves to reinforce the hypothesis that RB exerts an indirect immunological effect through the process of tumor cell death. Although RB exhibits intrinsic cytotoxic activity in the absence of light, its photoactivation introduces a distinct and complementary mechanism based on ROS generation. Illumination therefore does not amplify its baseline effect but enables spatially and temporally controlled oxidative damage within the illuminated area. This dual mechanism may allow effective tumor control at lower drug concentrations while potentially limiting systemic exposure. Clinical trials with cytotoxic intratumoral injection of RB are associated with systemic toxicity.[9,25]

While formal clinical comparisons between high-dose RB alone and low-dose light-activated RB remain to be conducted, our findings support the concept that photodynamic activation represents a mechanistically distinct therapeutic approach which would enable local anti-tumor treatments with less systemic toxicity.

The strengths of our studies are the comprehensive demonstration of optimization on RB in a therapeutic strategy combining low doses of RB with green light at 550 nm at 0,3J/cm^2, a specific wavelength that activates RB to achieve dynamic phototherapy for a targeted, less toxic, and immunogenic approach to cancer treatment, in line with current paradigms in cancer immunotherapy. We have combined the demonstration of a synergistic anti-tumor effect (40–121 time more efficient), confirmation of a PDT effect with ROS production and finally confirmed that this PDT-RB had a pro-immunogenic effect.

The use of 550 nm illumination was selected according to the photophysical properties of RB, which exhibits a strong absorption maximum in the 540–560 nm range associated with a high molar extinction coefficient and efficient singlet oxygen generation. Excitation at this wavelength therefore maximizes the photoactivation efficiency of the PS. Although melanin displays broad absorption across the visible spectrum, its absorption progressively decreases from the blue toward the red region, and reported light penetration at 550 nm remains compatible with the treatment of superficial lesions. Importantly, photodynamic efficacy results from the balance between tissue penetration and PS absorption efficiency. Shifting to longer wavelengths may increase penetration depth but would substantially reduce RB excitation and overall photodynamic yield. In this context, 550 nm illumination represents a rational and

mechanistically justified choice for superficial melanoma applications rather than an attempt to target deeply invasive tumors. Furthermore, early-stage skin melanoma metastatsis are mainly located in the epidermis and superficial dermis, which is consistent with this depth that could be targeted with PDT-RB.

A notable strength of our study is the integration of biological and immunological concepts with the precision of physics, leading to the development of illumination devices that enable precise, reproducible irradiation with dose escalation.[30] These technological devices facilitate precise responses to specific biological interrogations. Furthermore, collaboration with a physics team facilitates the development of illumination devices that enable the precise and optimized evaluation of PDT *in vivo*.[31] These devices can be adapted for human use in clinical trial conducted by our translational team, thus addressing the anatomical constraints that have been identified as a potential limitation to the application of PDT. These factors are encouraging with respect to the continuation of this *in vivo* project.

This study has several limitations. First, all experiments were conducted *in vitro*, and therefore do not fully recapitulate the complexity of tumor microenvironment, tissue optical properties, or metastatic dissemination observed *in vivo*.

The limitations of our study are that the present study was not designed to allow for the accurate identification of any differences in efficacy between the two cell lines. It is conceivable that these discrepancies can be attributed to variations in the cell proliferation process. Indeed, it has previously been observed that RB is capable of influencing the cell cycle.[21] Furthermore, the potential for disparities in the pigmentation process remains a subject of inquiry, despite the absence of substrates essential for melanoma pigmentation in the present study.[32] Finally, molecular differences between the two lines are possible.

Second, PDT is inherently limited by light penetration depth, which may restrict its application primarily to superficial or accessible lesions. Consequently, further *in vivo* investigations are necessary to evaluate treatment efficacy, biodistribution, and safety before considering potential clinical translation.

Finally, melanoma has undergone a real therapeutic revolution with the advent of targeted BRAF and MEK inhibitors and checkpoint inhibitors immunotherapies.[2] However, some patients present delayed responses or primary and/or secondary resistance. It would be interesting to evaluate the antitumor efficacy of PDT-RB on human melanoma cell lines resistant to these therapies. The antitumor and immunogenic effect of PDT-RB could make it an adjuvant treatment to conventional melanoma therapies.

In conclusion, our study demonstrates that photodynamic activation of RB enhances ROS-mediated cytotoxicity in melanoma cells *in vitro* and allows effective responses at lower drug concentrations. These findings provide a proof of concept supporting further evaluation of this strategy in more advanced preclinical models. Additional *in vivo* studies will be required to determine its therapeutic potential and clinical applicability.

MATERIALS AND METHODS

Reagents

For this study, we used the following reagent: RB (Sigma-Aldrich, St. louis, MO, USA). The RB powder (10% weigh/volume) was diluted in 0.9% NaCl to obtain the equivalent of PV-10. RB solutions were prepared fresh, protected from light, and maintained under dark conditions during incubation period. No visible color change or alteration in baseline fluorescence signal was observed over the experiment. CellTiter-Glo® viability kit (Promega, Madison, WI, USA), cytotoxicity detection kit (Roche, Bale, Switzerland), (methyl-^{3} H)-thymidine (PerkinElmer, Courtaboeuf, France), DCFDA (D6883, Sigma-Aldrich, St Louis, MO, USA), Hydrogen peroxide (H_2O_2) (H1009, Sigma-Aldrich, St Louis, MO, USA), paraformaldehyde (PAF) (Santa Cruz Biotechnology, Dallas, TX, USA), DAPI (Sigma-Aldrich, Saint-Louis, MO, USA).

Cell culture

The metastatic melanoma cell lines HBL and LND were given by Pr. Philippe Marchetti Team. HBL and LND were cultured in a Roswell Park Memorial Institute medium (RPMI 1640, Gibco Thermo Fisher Scientific, Waltham, MA, USA) supplemented with 100 units/mL penicillin, 100 g/mL streptomycin (Gibco Thermo Fisher Scientific, Waltham, MA, USA), 10% (v/v) of heat inactivated fetal bovine serum (FBS Gibco Thermo Fisher Scientific, Waltham, MA, USA) and maintained at 37°C in a humidified atmosphere with 5% CO_2.

Blood immune cells isolation

Human PBMCs were collected from healthy adult donors with informed consent, in accordance with the approval of the Institutional Review Board at the Biology Institute of Lille (DC-2013-1919). PBMCs were isolated by density gradient centrifugation of the blood using a lymphocyte separation medium (Eurobio, Les Ulis, France) and 50 mL Leucosep tubes (Greiner Bio One, Courtaboeuf, France). In round-bottom 96-well plate (Corning, Somerville, MA, USA) 100,000 PBMCs were cultured in ML10 medium composed of RPMI 1640 medium supplemented with HEPES (25 mM), non-essential amino acids (MEM 1*x*), sodium pyruvate (1 mM), 2-mercaptoethanol (50 μM), gentamicin (10 μg/mL) (Thermo Fisher Scientific, Waltham, MA, USA), and 10% of SAB (Human AB serum).

Incorporation study

Fluorimetry measurement

HBL and LND cells were seeded in 96 well culture plates (Corning, Somerville, MA, USA) at the concentration of 15,000 cells per wells and incubate for 24 h. Then cells were treated with increasing doses of RB (0, 5, 10, 25, 50, 75, 100, and 400 μM) and during several incubation time points (0, 0.5, 1, 2, 3, and 4 h). Throughout the experiment, the cells remained in total darkness. At the end of the incubation period, cells were washed twice with fresh media and new

medium was added to the cells to measure the intracellular levels of RB. RB fluorescence was measured by an excitation wavelength of 540 ± 15 nm and an emission wavelength of 570 ± 20 nm using CLARIOstar® Plus (BMG LABTECH, Ortenberg, Germany). The data were then interpreted using MARS software version 4.01 R2 (BMG LABTECH, Ortenberg, Germany). Results were expressed in relative fluorescence units (RFU) from 3 biological independent experiments in triplicate.

Confocal microscopy

30,000 cells were cultured on sterile 10 mm diameter coverslips in 12-well plates (Dutscher, France). 24 h after culture, medium was renewed with sterile medium containing 100 μM of RB for 2 h. Negative controls untreated were carried out. The cells were washed three times with PBS−/− and then fixed with a 4% paraformaldehyde (PAF) solution (Santa Cruz Biotechnology, Dallas, TX, USA). The cells were then incubated for 10 min with 50 ng/mL DAPI (Sigma-Aldrich, St. Louis, MO, USA) to obtain a nuclear stain and washed two times with PBS−/−. The coverslips containing the cells were then mounted on slides using Mowiol (Sigma-Aldrich, Saint-Louis, MO, USA) and stored at 4°C until analysis. The analysis was carried using a confocal microscope LSM 880 (Carl Zeiss, Oberkochen, Germany) with a magnification of 63×. The images were acquired by Zen Lite 2.3 (Zeiss, Oberkochen, Germany).

Cytotoxicity LDH

15,000 cells of HBL and LND cell lines were seeded in a 96-well plate (Corning, Somerville, MA, USA) in triplicate. 24 h after, medium was replaced by the aforementioned RB concentrations and incubation period used in the fluorometric assay experiments. Cells treated for 2 h with 2% Triton X-100 (Sigma-Aldrich, Saint-Louis, MO, USA) were considered as the positive control. After incubation medium was recover and place in a new 96 well plate (Corning, Somerville, MA, USA). The media was used immediately to analyze cytotoxicity by using a Cytotoxicity Detection kit (Roche, Sigma-Aldrich, Saint Louis, MO, USA). For that, 100 μL of the detection kit reagent was added to each well containing supernatant. Plates were incubated at room temperature in the dark for 20 min. After which an absorption measurement was performed at 492 nm using a CLARIOstar® Plus (BMG LABTECH, Ortenberg, Germany). Media without cells were used to serve as blank conditions. The data were then interpreted using MARS software version 4.01 R2 (BMG LABTECH, Ortenberg, Germany). The cytotoxicity was represented as relative cytotoxicity (%) = ([sample value − non-treated control]/[positive control − non-treated control]) × 100 from 3 biological independent experiments in triplicate.

Photodynamic therapy treatment

HBL and LND cells were seeded in 96-well culture plates with white walls and optically clear bottoms (Greiner Bio-One GMBH, Frickenhausen, Germany) at a concentration of 15,000 cells per well and incubated for 24 h. After 24 h, cancer cells were treated regarding different conditions: non-treated cell (NT), cells only illuminated (Illu), cells treated with RB only (RB) and cells treated with RB coupled with an illumination (PDT). For the RB treatment, 8 different concentrations of RB were used (0, 5, 10, 25, 50, 75, 100, and 400 μM) during 2 h before two washing steps using fresh medium and illumination treatment. Regarding the illumination process, we used the illumination device developed in our laboratory and previously described in the publication[30] with a final green (550 nm) light dose of 0.3 J/cm^2 with Cell-Led Device. The cells were then incubated in dark for 24 h. The concentrations inducing an 80% decrease in viability were then calculated for RB alone and under PDT-RB conditions. For each result 3 biological independent experiments were done in triplicate.

Viability assay

Melanoma cancer cell line viability after treatments was evaluated by a viability assay based on ATP release measurement by bioluminescence (CellTiterGlo, Promega, Madison, WI, USA). For that, 50 μL of CellTiter-Glo mix was added to each well and incubated for 10 min at room temperature, protected from light. The bioluminescence was then read by the CLARIOstar Plus (BMG LABTECH, Ortenberg, Germany) using SMART Control software version 6.20 (BMG LABTECH, Ortenberg, Germany). The data were then interpreted using MARS software version 4.01 R2 (BMG LABTECH, Ortenberg, Germany). Results were expressed in percentage from non-treated condition from 3 biological independent experiments in triplicate.

Production of post-PDT-RB supernatant

HBL and LND cells were seeded in 96-well culture plates with white walls and optically clear bottoms (Greiner Bio-One GMBH, Frickenhausen, Germany) at a concentration of 15,000 cells per well and incubated for 24 h. After 24 h, HBL and LND cells were treated in different conditions (NT: non treated, RB at EC80 dose [492 μM for HBL and 1,043 μM for LND], Illu: illumination only at 0.3 J/cm^2 and RB + light [PDT] [32 μM for HBL and 74 μM for LND]). 24 h after the treatment, medium of each condition was recover and preserved at −20°C until used for further experiments.

Ros production

Flow cytometry

Intracellular ROS production was quantified using the DCFDA cellular ROS detection assay". Cells were seeded in 25 cm^2 culture flasks and cultured for 24 h. Cells were then treated or not with RB at the EC80(RB) or EC80(PDT-RB) dose for 24 h. Cells were trypsinized and then labeled with 20 μM DCFDA (D6883, Sigma-Aldrich, St Louis, MO, USA) for 30 min at 37°C. DCFDA is deacetylated by cellular esterases to a non-fluorescent compound. This compound is subsequently oxidized by cellular ROS to form 2′,7′-dichlorofluorescein (DCF). DCF is a fluorescent compound that can be detected by flow cytometry (excitation: 485 nm; emission: 535 nm). Cells were then transferred to a 96-well plate, centrifuged at 1,000 rpm, 21°C for 5 min. Illumination as previously described. After treatment with illumination or addition of Hydrogen peroxide ([H_2O_2] = 4 mM) in positive control (H1009, Sigma-Aldrich, St Louis, MO, USA), cells were washed with PBS. Cellular ROS

generation was then quantified 20 min post-illumination using the Cytoflex (Beckman Coulter, Brea, CA, USA). Data were examined using Kaluza Analysis Software v.2.2.1 (Beckman Coulter Life Sciences, Brea, California, USA).

Fluorimetry measurement

Dose dependent ROS generation was analyzed quantified by fluorimetry. Cells were seeded in 96-well plates and cultured for 24 h. Cells were treated as previously described with increasing concentration of RB. After 2 h of incubation, cells were washed with 1× PBS and then labeled with 20 μM DCFDA for 30 min at 37°C. For positive control, cells were treated with H_2O_2 (0.1 mM) for 2 h after DCFDA labeling. Illumination was performed as previously described. Cellular ROS generation was then qualified immediately after illumination with CLARIOstar Plus (BMG LABTECH, Ortenberg, Germany) using SMART Control software version 6.20 (BMG LABTECH, Ortenberg, Germany).

Apoptosis/necrosis kenitic measurement

Melanoma cells lines were plates in 96-well culture plates with white walls and optically clear bottoms (Greiner Bio-One GMBH, Frickenhausen, Germany) at a concentration of 15,000 cells per well and incubated for 24 h. Then, cells were incubated 2 h with several RB concentrations (0, 10, EC50-PDT, EC80-PDT, and EC100-PDT and 400 μM) respectively (0, 10, 25, 32, 96, 400 μM) for HBL and (0, 10, 50, 72, 277, 400 μM) for LND. After cells were illuminated at the final light dose of 0.3 J/cm^2, then the RealTime-GloTM Annexin V Apoptosis and Necrosis Assay (JA1011, JA1012, Promega, USA) was add to the well according to the manufacturer protocol. Positive control of apoptosis was made by adding Staurosporine at 50 and 100 μM. Positive control of necrosis was made with Digitonin at 10 pg/mL. Finally, results of fluorescence and luminescence respectively necrosis and apoptosis signals were measured using the CLARIOstar Plus (BMG LABTECH, Ortenberg, Germany) using SMART Control software version 6.20 (BMG LABTECH, Ortenberg, Germany) for 60 h with a reading every 30 min. Staurosporine was used as a positive control of apoptosis and Digitonin as a positive control of necrosis (Figures S2A and S2B).

Proliferation assay

PBMCs were either stimulated or not stimulated with anti-CD3 (0.25 μg/mL; Miltenyi, Bergisch Glad bach, Germany) and anti-CD28 (0.25 μg/mL; Clinisciences, Montrouge, France). 50 μL of melanoma cells conditioned media was added to PBMCs in 96 round bottom plates during several incubation time (72 h–120 h).

Proliferation assays measured by adding radioactive [3H] thymidine (1μCi/well) (PerkinElmer, Courtaboeuf, France) to each well 18 h before harvesting. At the end of the culture, the cells were harvested on a glass fiber filter (PerkinElmer, Courtaboeuf, France) using a Tomtec harvester (Wallac, Turku, Finland), then sealed in a sample bag (PerkinElmer, Courtaboeuf, France) with scintillation liquid (Beckman Coulter, Brea, CA, USA). Radioactive thymidine was measured by scintillation counting using a β-counter (1450 Trilux, Wallac, Finland). Proliferation was estimated in count per minute (CPM) and expressed in CPM or normalized CPM. Normalized CPM = CPM of the sample/Average CPM of the control condition.

For normalization, the mean value of the non-treated (NT) group was first calculated from independent replicates. All experimental conditions, including individual NT replicates, were then normalized to this mean value. Because normalization was performed using the group mean rather than assigning a fixed value of 1 to each NT replicate; variability within the NT group was preserved and is therefore reflected by the corresponding standard deviation.

Statistical analysis

All experiments were performed with at least three independent biological replicates. Data are presented as mean ± standard error of the mean (SEM). For normalization, the mean value of the non-treated (NT) group was calculated and used as reference. All conditions, including individual NT replicates, were normalized to this mean value, thereby preserving variability within the NT group. Statistical analyses were performed using GraphPad Prism (GraphPad Software, USA). Comparisons between groups were carried out using one-way ANOVA followed by appropriate post hoc tests, as indicated in the figure legends. A p value <0.05 was considered statistically significant.

DATA AND CODE AVAILABILITY

Datasets related to this article can be found at upon request.

ACKNOWLEDGMENTS

The authors would like to thank the BioImaging Center Lille (“Plateformes Lilloises en Biologie et Santé (PLBS) - UAR 2014 - US 41”) for access to flow cytometry equipment. We would like to thank GEFLUC and the Bioderma Foundation for the award for the best photobiology posters at the conference. We would like to thank Philip Marchetti’s team for providing us with the HBL and LND cells.

AUTHOR CONTRIBUTIONS

Conceptualization, N.D.; data curation, M.B., A.L., M.K.M., and S.M.; formal analysis, M.B. and A.L.; funding acquisition, N.D. and L.M.; investigation, M.B. and A.L.; methodology, M.B., A.L., G.P.G., P.D., A.-S.D., O.M., N.D.; project administration, N.D. and L.M.; resources, N.D. and L.M.; supervision, G.P.G., A.-S.D., O.M., N.D., and L.M.; validation, all authors; visualization, M.B. and A.L.; writing – original draft, M.B. and A.L.; writing – review and editing, all authors.

DECLARATION OF INTERESTS

The authors declare no conflict of interest.

SUPPLEMENTAL INFORMATION

Supplemental information can be found online at https://doi.org/10.1016/j.omton.2026.201241.

REFERENCES

1. Dousset, L., Poizeau, F., Robert, C., Mansard, S., Mortier, L., Caumont, C., Routier, É., Dupuy, A., Rouanet, J., Battistella, M., et al. (2021). Positive Association between Location of Melanoma, Ultraviolet Signature, Tumor Mutational Burden, and Response to Anti–PD-1 Therapy (JCO Precision Oncology), pp. 1821–1829. https://doi.org/10.1200/PO.21.00084.

2. Garbe, C., Amaral, T., Peris, K., Hauschild, A., Arenberger, P., Basset-Seguin, N., Bastholt, L., Bataille, V., del Marmol, V., Dréno, B., et al. (2022). European

consensus-based interdisciplinary guideline for melanoma. Part 2: Treatment - Update 2022. Eur. J. Cancer *170*, 256–284. https://doi.org/10.1016/j.ejca.2022.04.018.

3. Weber, J.S., Carlino, M.S., Khattak, A., Meniawy, T., Ansstas, G., Taylor, M.H., Kim, K.B., McKean, M., Long, G.V., Sullivan, R.J., et al. (2024). Individualised neoantigen therapy mRNA-4157 (V940) plus pembrolizumab versus pembrolizumab monotherapy in resected melanoma (KEYNOTE-942): a randomised, phase 2b study. Lancet *403*, 632–644. https://doi.org/10.1016/S0140-6736(23)02268-7.

4. Demartis, S., Obinu, A., Gavini, E., Giunchedi, P., and Rassu, G. (2021). Nanotechnology-based rose Bengal: A broad-spectrum biomedical tool. Dyes and Pigments *188*, 109236. https://doi.org/10.1016/j.dyepig.2021.109236.

5. Thompson, J.F., Hersey, P., and Wachter, E. (2008). Chemoablation of metastatic melanoma using intralesional Rose Bengal. Melanoma Res. *18*, 405–411. https://doi.org/10.1097/CMR.0b013e32831328c7.

6. Thompson, J.F., Agarwala, S.S., Smithers, B.M., Ross, M.I., Scoggins, C.R., Coventry, B.J., Neuhaus, S.J., Minor, D.R., Singer, J.M., and Wachter, E.A. (2015). Phase 2 Study of Intralesional PV-10 in Refractory Metastatic Melanoma. Ann. Surg. Oncol. *22*, 2135–2142. https://doi.org/10.1245/s10434-014-4169-5.

7. Read, T., Fayers, W., Thomas, J., Wagels, M., Barbour, A., and Mark Smithers, B. (2019). Patients with in-transit melanoma metastases have comparable survival outcomes following isolated limb infusion or intralesional PV-10—A propensity score matched, single center study. J. Surg. Oncol. *119*, 717–727. https://doi.org/10.1002/jso.25373.

8. Read, T.A., Smith, A., Thomas, J., David, M., Foote, M., Wagels, M., Barbour, A., and Smithers, B.M. (2018). Intralesional PV-10 for the treatment of in-transit melanoma metastases—Results of a prospective, non-randomized, single center study. J. Surg. Oncol. *117*, 579–587. https://doi.org/10.1002/jso.24921.

9. Wiener, M., Damian, D.L., and Thompson, J.F. (2008). Systemic Phototoxicity following Intralesional Rose Bengal for Subcutaneous Melanoma Metastases. Dermatology *216*, 361–362. https://doi.org/10.1159/000117707.

10. Falk-Mahapatra, R., and Gollnick, S.O. (2020). Photodynamic Therapy and Immunity: An Update. Photochem. Photobiol. *96*, 550–559. https://doi.org/10.1111/php.13253.

11. Trioën, C., Soulier, T., Massoud, J., Bouchez, C., Stoup, N., Lefebvre, A., Dewalle, A.-S., Grolez, G.P., Delhem, N., and Moralès, O. (2025). Efficient 5-ALA-photodynamic therapy in nasopharyngeal carcinoma induces an immunoactivation mediated by tumoral extracellular vesicles and associated with immunogenic cell death. Cancer Commun. *45*, 401–405. https://doi.org/10.1002/cac2.12656.

12. Kwiatkowski, S., Knap, B., Przystupski, D., Saczko, J., Kędzierska, E., Knap-Czop, K., Kotlińska, J., Michel, O., Kotowski, K., and Kulbacka, J. (2018). Photodynamic therapy - mechanisms, photosensitizers and combinations. Biomed. Pharmacother. *106*, 1098–1107. https://doi.org/10.1016/j.biopha.2018.07.049.

13. Morton, C.A., Szeimies, R.-M., Basset-Seguin, N., Calzavara-Pinton, P., Gilaberte, Y., Hædersdal, M., Hofbauer, G.F.L., Hunger, R.E., Karrer, S., Piaserico, S., et al. (2019). European Dermatology Forum guidelines on topical photodynamic therapy 2019 Part 1: treatment delivery and established indications – actinic keratoses, Bowen's disease and basal cell carcinomas. J. Eur. Acad. Dermatol. Venereol. *33*, 2225–2238. https://doi.org/10.1111/jdv.16017.

14. Yan, E., Kwek, G., Qing, N.S., Lingesh, S., and Xing, B. (2023). Antimicrobial Photodynamic Therapy for the Remote Eradication of Bacteria. Chempluschem *88*, e202300009. https://doi.org/10.1002/cplu.202300009.

15. Ren, F., Zhao, S., Yang, C., Liu, J., Shang, Q., Feng, K., Kang, X., Zhang, R., Wang, X., and Wang, X. (2023). Applications of photodynamic therapy in extramammary Paget's disease. Am. J. Cancer Res. *13*, 4492–4507.

16. Mazur, E., Kwiatkowska, D., and Reich, A. (2023). Photodynamic Therapy in Pigmented Basal Cell Carcinoma-A Review. Biomedicines *11*, 3099. https://doi.org/10.3390/biomedicines11113099.

17. Baldea, I., Giurgiu, L., Teacoe, I.D., Olteanu, D.E., Olteanu, F.C., Clichici, S., and Filip, G.A. (2019). Photodynamic Therapy in Melanoma - Where do we Stand? Curr. Med. Chem. *25*, 5540–5563. https://doi.org/10.2174/0929867325666171226115626.

18. Dhillon, S.K., Porter, S.L., Rizk, N., Sheng, Y., McKaig, T., Burnett, K., White, B., Nesbitt, H., Matin, R.N., McHale, A.P., et al. (2020). Rose Bengal–Amphiphilic Peptide Conjugate for Enhanced Photodynamic Therapy of Malignant Melanoma. J. Med. Chem. *63*, 1328–1336. https://doi.org/10.1021/acs.jmedchem.9b01802.

19. Gianotti, E., Martins Estevão, B., Cucinotta, F., Hioka, N., Rizzi, M., Renò, F., and Marchese, L. (2014). An Efficient Rose Bengal Based Nanoplatform for Photodynamic Therapy. Chem. Eur J. *20*, 10921–10925. https://doi.org/10.1002/chem.201404296.

20. Toomey, P., Kodumudi, K., Weber, A., Kuhn, L., Moore, E., Sarnaik, A.A., and Pilon-Thomas, S. (2013). Intralesional injection of rose bengal induces a systemic tumor-specific immune response in murine models of melanoma and breast cancer. PLoS One *8*, e68561. https://doi.org/10.1371/journal.pone.0068561.

21. Qin, J., Kunda, N., Qiao, G., Calata, J.F., Pardiwala, K., Prabhakar, B.S., and Maker, A.V. (2017). Colon cancer cell treatment with rose bengal generates a protective immune response via immunogenic cell death. Cell Death Dis. *8*, e2584. https://doi.org/10.1038/cddis.2016.473.

22. Liu, H., Innamarato, P.P., Kodumudi, K., Weber, A., Nemoto, S., Robinson, J.L., Crago, G., McCardle, T., Royster, E., Sarnaik, A.A., and Pilon-Thomas, S. (2016). Intralesional rose bengal in melanoma elicits tumor immunity via activation of dendritic cells by the release of high mobility group box 1. Oncotarget *7*, 37893–37905. https://doi.org/10.18632/oncotarget.9247.

23. Zhang, L., Du, J., Song, Q., Zhang, C., and Wu, X. (2022). A Novel In Situ Dendritic Cell Vaccine Triggered by Rose Bengal Enhances Adaptive Antitumour Immunity. J. Immunol. Res. *2022*, 1178874. https://doi.org/10.1155/2022/1178874.

24. Ross, M.I. (2014). Intralesional therapy with PV-10 (Rose Bengal) for in-transit melanoma. J. Surg. Oncol. *109*, 314–319. https://doi.org/10.1002/jso.23554.

25. Patel, S.P., Carter, B.W., Murthy, R., Sheth, R., Agarwala, S.S., Lu, G., Redstone, E., Balmes, G.C., Rider, H., Rodrigues, D., and Wachter, E.A. (2020). Percutaneous hepatic injection of rose bengal disodium (PV-10) in metastatic uveal melanoma. J. Clin. Oncol. *38*, 3143. https://doi.org/10.1200/JCO.2020.38.15_suppl.3143.

26. Panzarini, E., Inguscio, V., and Dini, L. (2011). Overview of Cell Death Mechanisms Induced by Rose Bengal Acetate-Photodynamic Therapy. Int. J. Photoenergy *2011*, 1–11. https://doi.org/10.1155/2011/713726.

27. Panzarini, E., Inguscio, V., Fimia, G.M., and Dini, L. (2014). Rose Bengal acetate photodynamic therapy (RBAc-PDT) induces exposure and release of Damage-Associated Molecular Patterns (DAMPs) in human HeLa cells. PLoS One *9*, e105778. https://doi.org/10.1371/journal.pone.0105778.

28. Srivastav, A.K., Mujtaba, S.F., Dwivedi, A., Amar, S.K., Goyal, S., Verma, A., Kushwaha, H.N., Chaturvedi, R.K., and Ray, R.S. (2016). Photosensitized rose Bengal-induced phototoxicity on human melanoma cell line under natural sunlight exposure. J. Photochem. Photobiol. B *156*, 87–99. https://doi.org/10.1016/j.jphotobiol.2015.12.001.

29. Mousavi, H., Zhang, X., Gillespie, S., Wachter, E., and Hersey, P. (2006). Rose Bengal induces dual modes of cell death in melanoma cells and has clinical activity against melanoma. Melanoma Res. *16*, S8.

30. Lefebvre, A., Marhfor, S., Baert, G., Deleporte, P., Grolez, G.P., Boileau, M., Morales, O., Vignoud, S., Delhem, N., Mortier, L., and Dewalle, A.S. (2024). Photodynamic Therapy Using a Rose-Bengal Photosensitizer for Hepatocellular Carcinoma Treatment: Proposition for a Novel Green LED-Based Device for In Vitro Investigation. Biomedicines *12*, 2120. https://doi.org/10.3390/biomedicines12092120.

31. Baydoun, M., Boidin, L., Leroux, B., Vignion-Dewalle, A.-S., Quilbe, A., Grolez, G.P., Azaïs, H., Frochot, C., Moralès, O., and Delhem, N. (2023). Folate Receptor Targeted Photodynamic Therapy: A Novel Way to Stimulate Anti-Tumor Immune Response in Intraperitoneal Ovarian Cancer. Int. J. Mol. Sci. *24*, 11288. https://doi.org/10.3390/ijms241411288.

32. Kalegari, P., Leme, D.M., Disner, G.R., Cestari, M.M., de Lima Bellan, D., Meira, W.V., Mazepa, E., and Martinez, G.R. (2022). High Melanin Content in Melanoma Cells Contributes to Enhanced DNA Damage after Rose Bengal Photosensitization. Photochem. Photobiol. *98*, 1355–1364. https://doi.org/10.1111/php.13632.

OMTON, Volume *34*

Supplemental information

Innovative photodynamic therapy using rose bengal for the treatment of human melanoma

Marie Boileau, Anthony Lefebvre, Smail Marhfor, Meriem Kali Mansouri, Pascal Deleporte, Guillaume Paul Grolez, Anne-Sophie Dewalle, Olivier Morales, Nadira Delhem, and Laurent Mortier

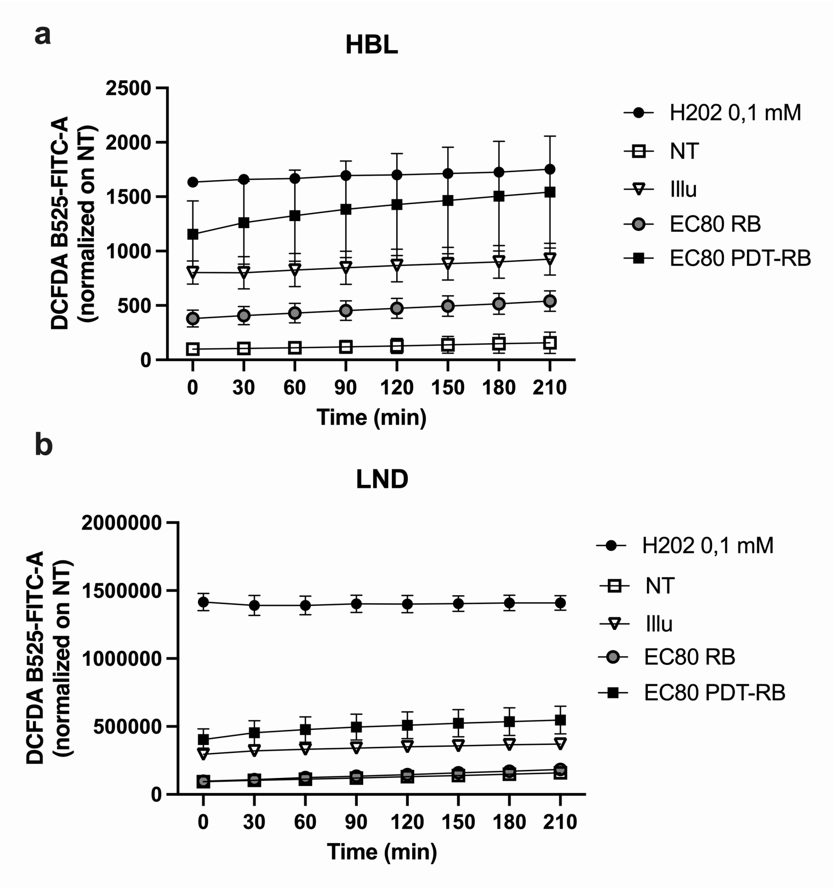

Figure S1

ROS production kinetics were assessed every 30 minutes over a period of 3.5 hours **(a for HBL and b for LND).** ROS production was constant over time. NT: Non treated cells, Illu: illuminated alone without Rose Bengal, RB: Rose Bengal, PDT-RB: cells incubated with RB and illuminated, H2O2 : positive control

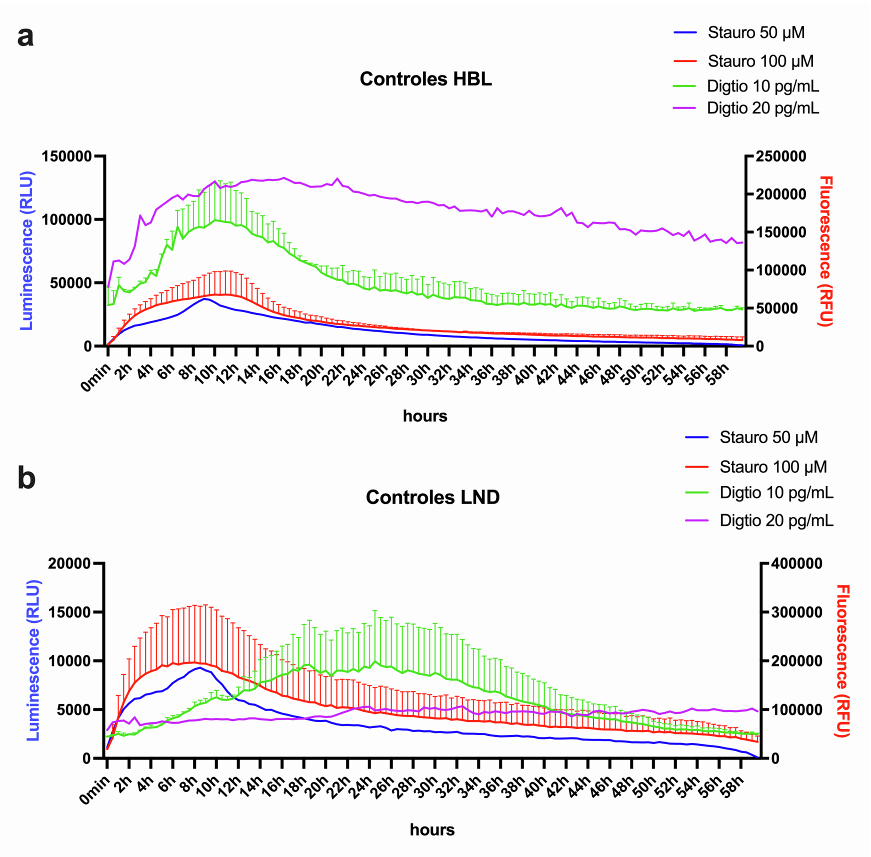

Figure S2

Kinetic profiles of Phosphatidyl Serine exposure reflecting apoptosis (luminescence) and loss of membrane integrity reflecting necrosis (fluorescence) over time according to the concentration of RB. Luminescence and Fluorescence signal were collected by sequential measurements of the same plate over the 60h time course. Staurosporine was used as a positive control of apoptosis and Digitonin as a positive control of necrosis **(a for HBL and b for LND)**
